# Defensome against Toxic Diatom Aldehydes in the Sea Urchin *Paracentrotus lividus*

**DOI:** 10.1371/journal.pone.0031750

**Published:** 2012-02-20

**Authors:** Vincenzo Marrone, Marina Piscopo, Giovanna Romano, Adrianna Ianora, Anna Palumbo, Maria Costantini

**Affiliations:** 1 Laboratory of Cellular and Developmental Biology, Stazione Zoologica Anton Dohrn, Naples, Italy; 2 Department of Structural and Functional Biology, University of Naples Federico II, Naples, Italy; 3 Laboratory of Functional and Evolutionary Ecology, Stazione Zoologica Anton Dohrn, Naples, Italy; Ecole Normale Supérieure de Lyon, France

## Abstract

Many diatom species produce polyunsaturated aldehydes, such as decadienal, which compromise embryonic and larval development in benthic organisms. Here newly fertilized *Paracentrotus lividus* sea urchins were exposed to low concentration of decadienal and the expression levels of sixteen genes, implicated in a broad range of functional responses, were followed by Real Time qPCR in order to identify potential decadienal targets. We show that at low decadienal concentrations the sea urchin *Paracentrotus lividus* places in motion different classes of genes to defend itself against this toxic aldehyde, activating *hsp60* and two proteases, *hat* and *BP10*, at the blastula stage and *hsp56* and several other genes (*14-3-3ε*, *p38 MAPK*, *MTase*, and *GS*) at the prism stage. At this latter stage all genes involved in skeletogenesis (*Nec*, *uni*, *SM50* and *SM30*) were also down-expressed, following developmental abnormalities that mainly affected skeleton morphogenesis. Moreover, sea urchin embryos treated with increasing concentrations of decadienal revealed a dose-dependent response of activated target genes. Finally, we suggest that this orchestrated defense system against decadienal represents part of the chemical defensome of *P. lividus* affording protection from environmental toxicants.

## Introduction

The sea urchin is considered a good model species to study the ecotoxicological response of marine invertebrates to environmental pollutants. It is world-wide in distribution and important in structuring benthic marine communities. Maintenance of these animals and gamete preparation are relatively easy, development is sensitive to several kinds of pollutants, and results can be obtained in a short period of time [1]–[3]. The transparent embryo is suitable for the observation of malformation, making it possible to detect sub-lethal effects of pollutants on multicellular body formation at an early stage in development. To date the stressors that have been examined, using sea urchin as a model, include physical changes of the water milieu, such as acidic pH [4], hypoxia [5] and X-rays [6], [7], and chemicals such as antifouling agents/pesticides [8], [9], endocrine disrupter compounds [10], [11] and heavy metals [3], [12], [13]. Natural toxins can also represent a major source of stress for marine organisms. Of particular note are algal neurotoxins that can cause mass mortalities in fish, sea birds and marine mammals, and cytotoxic compounds such as the polyunsaturated aldehydes (PUAs) that can induce reproductive failure in some predatory crustacean copepods and other invertebrates [14], [15]. For example, the diatom-derived PUA decadienal has been shown to have deleterious (teratogenic) effects on embryonic and larval development of sea urchins even at low doses [16].

Moreover, Romano et al. [17] reported that treatment of sea urchin *Paracentrotus lividus* embryos with decadienal provokes nitric oxide-mediated activation of heat shock protein 70 in order to protect developing embryos against teratogenesis.

Previous reports have shown that HSP60 protein levels increase after heat shock or cadmium exposure in *P. lividus* embryos [18]. HSP70, generally used for the assessment of vertebrate cellular health state [19] and tumor occurrence [20], has been recognized as a valid biomarker of exposure to pollutants and UV-B radiation in embryos, as well as in adult immune cells of the sea urchin [18], [21]–[26] and it is also well known that the *hsp70* gene is a sensitive marker of stress. Both vertebrates and invertebrates overexpress the HSP70 group of proteins in response to a wide variety of natural, experimental or anthropogenic stressors [27]–[29], as protective agents in the acquisition of tolerance and resistance to apoptosis.

Here we further investigate the molecular basis of the stress response of sea urchin embryos to PUAs. To this aim we first treated sea urchin embryos with a low concentration of decadienal and followed by Real Time qPCR the expression levels of sixteen genes, in order to identify genes that were activated in response to this teratogen. Moreover, we treated embryos with increasing concentrations of decadienal to reveal a dose-dependent response of activated genes. Morphological analysis was also carried out during embryonic development to correlate teratogenic changes with gene expression patterns.

## Results

### Gene tress gene response to decadienal-induced teratogenesis

As shown in a previous study [17], teratogenesis in the sea urchin *P. lividus* occurs at >0.2 µg/ml decadienal concentration with an increase in the number of abnormal plutei. Such plutei showed severe malformations such as asymmetrical arms and spicules, reduced length of the arms and spicules, and a shortening of the apex as if retarded in growth. Moreover, Romano et al. [17] showed that 0.25 µg/ml decadienal represented the best concentration to simultaneously study decadienal-induced morphological effects and gene expression response. To better understand these effects at the molecular level, *P. lividus* embryos were incubated for 10 minutes in 0.25 µg/ml decadienal and samples were collected at 5, 9, 24 and 48 hours post fertilization (hpf), corresponding to the stages of early blastula, swimming blastula, prism and pluteus. We then followed by Real Time qPCR the expression levels of sixteen genes, implicated in various functional responses in sea urchins including stress, development, hatching and skeletogenesis (see Table S1). Our control gene for Real Time qPCR was ubiquitin, the expression of which remained constant in all sea urchin developmental stages.

**Figure 1:**
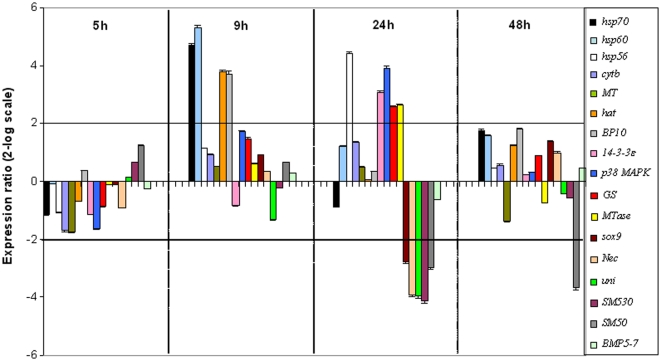
Gene activation in response to a low concentrations of decadienal. Histogram shows the differences in expression levels of analyzed stress genes, followed by Real Time qPCR. Embryos incubated with decadienal 0.25 µg/ml were collected at 5, 9, 24 and 48 hpf. Data are reported as a fold difference compared to control (mean ± SD), embryos in sea water without decadienal. Fold differences greater than ±2 (see dotted horizontal guidelines at values of 2 and −2) were considered significant. (For more details on genes see also Table 1 and Table S1; see also Materials and Methods for Real Time qPCR).

The histogram reported in Figure 1 shows the relative expression ratios of the analyzed genes with respect to the control, embryos in sea water without decadienal. Only expression values greater than a 2-fold difference over controls were considered significant. We also reported the expression level of *hsp70*, previously shown to be upregulated in response to decadienal stress in Romano et al. [17]. At early blastula stage (5 hpf) the expression levels of all genes remained at the basal levels and were comparable to the control. At swimming blastula stage (9 hpf) we found an increase in the expression levels of *hsp60*, *hat* and *BP10*, which showed a 5.3-, 3.8- and 3.7-fold increase with respect to the control, respectively. At prism stage (24 hpf) other genes were activated: *hsp56*, *14-3-3ε*, *p38 MAPK*, *GS* and *MTase* genes, which showed a 4.4-, 3.1-, 4.0-, 2.5- and 2.6- fold increase with respect to the control, respectively. At this stage of development, we also observed a 2.8-, 3.9-, 3.9-, 4.1- and 3.0- fold down-regulation of s*ox9*, *Nec*, *uni*, *SM30* and *SM50* genes, respectively. Only the skeletogenic gene *SM50* showed a 3.7-fold decrease in expression level at the pluteus stage (48 hpf). The exact values shown in this figure are reported in Table S2.

### Dose-dependence effects of decadienal on gene expression

In a new set of experiments developing embryos of *P. lividus* were incubated in the presence of increasing decadienal concentrations (0.15, 0.20, 0.25, 0.30, 0.35 µg/ml) and samples were collected at 5, 9, 24 and 48 hpf.

**Figure 2:**
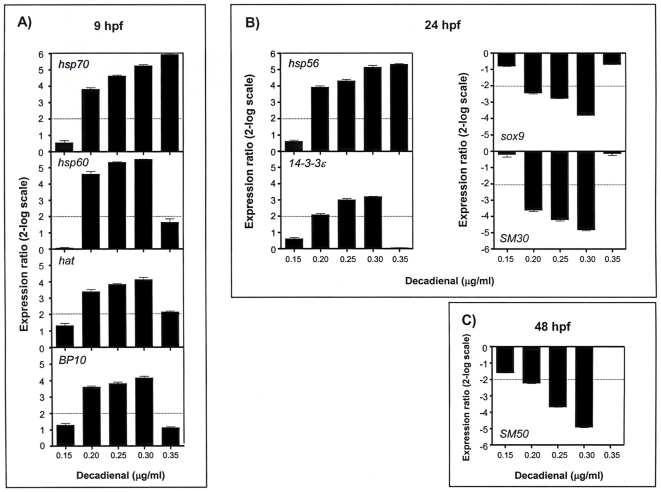
Dose-dependence effects of decadienal on gene expression. Histograms show decadienal dose-dependent variations in expression levels. Samples incubated with increasing decadienal concentrations (0.15, 0.20, 0.25, 0.30, 0.35 µg/ml) were collected at different times of development. A) decadienal dose-dependent overexpression for *hsp70*, *hsp60*, *hat* and *BP10* genes at 9 hpf; B) overexpression of *hsp56* and *14-3-3ε* and dow-expression of skeletogenic genes *sox9* and *SM30* at 24 hpf; C) down-expression of *SM50* at 48 hpf. (For more details see Legend to Figure 1).

A decadienal dose- and stage-dependent effect was detected by Real Time qPCR at the gene level for most of the analysed genes (Figure 2). At low decadienal concentrations (0.15 µg/ml) there was no gene stress response whereas at somewhat higher concentrations (0.20 µg/ml) a first series of genes were activated. The relative expression ratios of the analyzed genes with respect to the control, embryos in sea water without decadienal, are reported. Changes in gene expression were considered significant only at greater than a 2 fold level over controls. In particular, we observed an increase in the expression levels at swimming blastula stage (9 hpf) for all four genes switched on at low decadienal concentration: *hsp70* (from decadienal 0.20 to 0.35 µg/ml), *hsp60* (from 0.20 to 0.30 µg/ml), *hat* (from 0.20 to 0.30 µg/ml) and *BP10* (from 0.20 to 0.30 µg/ml) (see Figure 2A). Whereas at the prism stage (24hpf) dose-dependent increase in expression levels was found for *hsp56* (from 0.20 to 0.35 µg/ml) and *14-3-3ε* (from 0.20 to 0.30 µg/ml); a decrease was recorded for *sox9* (from 0.20 to 0.30 µg/ml decadienal) and *SM30* (from 0.20 to 0.30 µg/ml) genes (see Figure 2B). The *SM50* gene revealed a very strong dose-dependent decrease in its expression level at pluteus stage (48 hpf; from 0.20 to 0.30 µg/ml decadienal; see Figure 2C).

Interestingly, some genes that remained at the basal level at a low decadienal concentrations (0.25 µg/ml; see also Figure 1 and Table S2) were activated at higher decadienal concentrations (in Table S3 see for example *MT* that showed a 2.7-fold increase at 0.30 µg/ml decadienal and *cytb* a 2.3-fold increase at 0.35 µg/ml decadienal).

**Figure 3:**
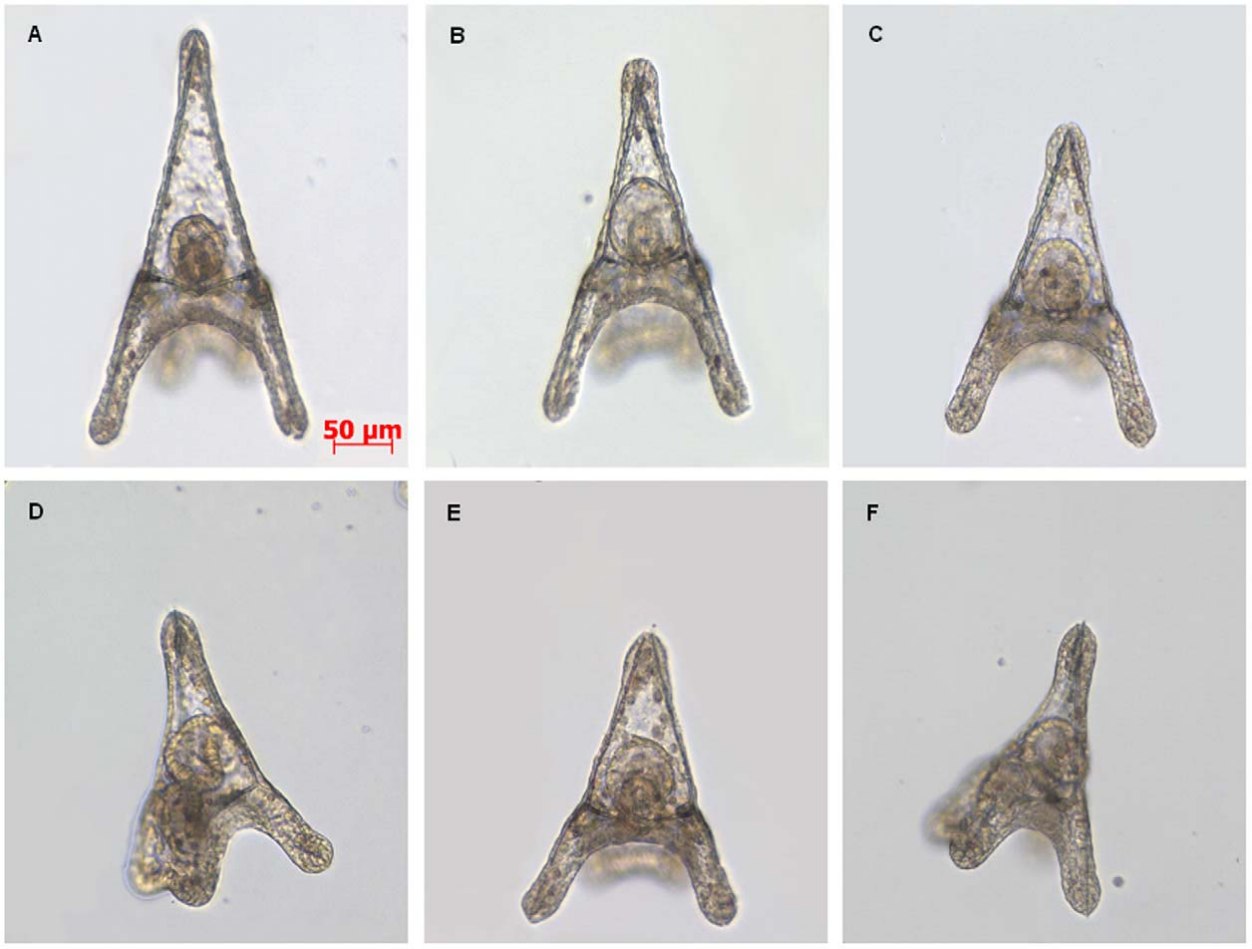
Dose-dependence effects of decadienal on sea urchin morphogenesis. (A) Control (embryos in sea water without decadienal), length 364.3 µm. (B) decadienal 0.15 µg/ml, length 317.6 µm, (C) decadienal 0.20 µg/ml, length 288.3 µm, (D) decadienal 0.25 µg/ml, length 261.6 µm, (E) 0.30 µg/ml, length 260.3 µm, (F) decadienal 0.35 µg/ml, length 253.6 µm. The images were taken at 48 hpf.

We also monitored the presence of morphological abnormalities by microscopic inspection 48 hours post treatment. In accordance with our previous data we found a dose-dependent increase in the number of abnormal embryos with increasing decadienal concentrations [16], [17]. In addition, we report, for the range of decadienal concentrations tested in the present study, a dose-dependent delay in the development of embryos, manifested by a shortening of the apex and arms (Figure 3). At 0.15 µg/ml the morphology of the embryo closely resembles that of control and only a slight reduction of body length can be observed. At decadienal concentrations ≥0.20 µg/ml the shortening of the body is more pronounced and the morphology of the embryo is altered, as revealed by the disorganization of epidermal cells at the tip of the arms.

## Discussion

The results reported in this work greatly expand our previous investigations [16], [17] on the stress response to the toxic PUA decadienal during sea urchin development. In addition to *hsp70*, whose expression was recently shown to be modulated by decadienal treatment at 0.25 µg/ml [17], we here report on the activity of a series of other genes that are responsive to decadienal.

At the early blastula stage (5 hpf) there was no significant activation of genes in response to low decadienal treatment, easily explained by the fact that it is too soon for sea urchin to counteract this injury. On the other hand, at the swimming blastula stage (9 hpf) we recorded the activation of *hsp60* and of two proteases, *hat* and *BP10*. Several studies in higher invertebrates and vertebrates have reported the activation of protection systems by increasing the expression of metal binding proteins [30] and heat shock proteins [18], [31] when exposed to heavy metals. Heat shock proteins play a critical role in a complex defense mechanism, by enhancing cell survival under adverse environmental conditions as well as in normal cellular homeostasis [32]. In fact, such proteins are capable of functioning as molecular chaperones by participating in protein synthesis and maturation, folding, assembly and disassembly of protein complexes, proteolysis and intracellular trafficking, thereby affecting the activity of key regulatory protein activity, cell proliferation, stress resistance, and apoptosis [33], [34]. Previous reports have shown that hsp60 and hsp56 protein levels increase after heat shock, manganese and cadmium exposure in *P. lividus* embryos [3], [7], [18], [35]. Both *hat* and *BP10* are early embryonic messengers, transiently expressed during the blastula stage [36]–[38]. A noteworthy observation from this study is the increased expression level of these proteases, when both genes are expressed. Further studies will be necessary to better understand the biological relevance of this increase.

At the prism stage (24 hpf), we found the upregulation of another heat shock protein, *hsp56*, known to be activated under similar stress conditions as hsp60 [18], [35] and several other genes *14-3-3ε*, *p38 MAPK*, *MTase*, and *GS*. 14-3-3 proteins are a family of regulatory molecules able to bind functionally diverse signaling proteins, such as kinases and phosphatases [39], whereas p38 MAPK [21] are involved in cell differentiation, survival and apoptosis, as well as participating in a signaling cascade in response to different stress stimuli. Several papers have reported that UVB radiation stimulates the expression of members of the 14-3-3ε protein family [39] and p38 MAPK [21], consistent with their role in mediating cellular response to stress and suggesting a function in embryo survival. The increase in expression level of *MTase* in response to decadienal injury represents an interesting result, closely related to evidence that sheds new light on the possible role of DNA methylation as a molecular marker in response to stress [40]. Chromatin remodeling has been shown to play a key role in the transcriptional activation of regulatory factors in response to a variety of stress signals [41]. The finding that *GS* is induced by decadienal treatment provides the first demonstration of the involvement of this gene in the stress response in sea urchin embryos, in line with some studies in plants [42], [43]. The induction of *MT* expression requires higher concentrations of decadienal, probably depending on the nature of the stress agent. Indeed, metallotionein has been reported to be activated in sea urchin embryos by cadmium treatment [30]. Also the expression level of *cytb* is slightly affected at higher concentrations of decadienal (0.35 µg/ml), but in the literature there are no reports on the stress response of this gene.

An important outcome of this study is the finding that the developmental abnormalities following decadienal treatment mainly affected skeleton morphogenesis as revealed by a shortening of the apex and arms. In accordance with these data, we observed a down-expression of all the genes involved during skeletogenesis at the prism stage, including *Nec*, *uni*, *SM50* and *SM30*), when all skeleton structures are well established. The expression levels of some of these skeletogenic genes have been shown to be affected also by manganese [44] and by X-rays [25]. In this context, a model has been proposed whereby some ectodermal cells secrete processed univin or a related factor into the blastocoel, where it signals primary mesenchyme cells to synthesize specific matrix proteins, such as SM30 and SM50 [45]. The ability of these ectodermal cells to produce this signal depends on their association with nectin [46] in the apical extracellular matrix. In our study the *SM50* gene was still down-regulated at the pluteus stage, in accordance with morphological observations that, at this time of development, the majority of embryos failed to reach the pluteus stage. Our finding that even at higher decadienal concentrations the expression level of growth factor *BMP5-7* was not affected (see Table S2) is in line with previous data, in which it has been shown that this gene is not involved during skeletogenesis [45]. Another developmental gene, *sox9*, involved in the left-right asymmetry process [47], was also down-regulated at the prism stage after decadienal treatment. According to our data, we suggest that decadienal affects the majority of genes at the swimming blastula and prism stages, in accordance with the fact that during these two developmental stages the vast majority of morphological processes occur. From the prism to pluteus stages embryos are fully formed and should only supplement growth. Moreover, our results clearly indicate that decadienal has a very broad spectrum of target genes, ranging from canonical stress genes to developmental and skeletogenic genes (see also Table S1).

However, decadienal is not only capable of switching on its target genes at certain concentrations, depending on gene sensitivity, but its mechanism of action seems to be highly sophisticated. In fact, in our study we demonstrate a decadienal dose-dependent effect on the expression of most genes, already switched on at low concentrations (0.2–0.3 µg/ml), while the percentage of abnormal nauplii is still low [16], [17]. Moreover, some genes, such as the heat shock proteins *hsp70* and *hsp56*, were more sensitive to decadienal than others, showing an increase in expression levels at the highest concentrations tested (see Table S3). Other genes (such as *hsp60*, *hat* and *BP10* a 9 hpf, *14-3-3ε*, *sox9* and *SM30* at 24 hpf, and *SM50* at 48 hpf) showed a decadienal dose-dependence increase until a concentration of 0.30 µg/ml, as if they can do nothing further to protect the embryos beyond this concentration and therefore yield to stress. These data suggest a very subtle adjustment to decadienal during the developmental process of sea urchin embryos.

In conclusion, in our study we demonstrate that *P. lividus* places in motion different classes of genes, in order to defend itself against this toxic diatom aldehyde. These genes could represent general biomarkers to detect exposure to pollutants, in agreement with some previous data reported on *Strongylocentrotus purpuratus*. In fact, the need to deal with physical, chemical, and biological challenges has driven the evolution of an array of gene families and pathways affording protection from, and repair of, damage to stress. Genes and proteins affording such protection for an organism collectively may be considered a “defensome”, as reported for the sea urchin *Strongylocentrotus purpuratus* [48]. A central part of this system is the “chemical defensome”, represented by an integrated network of genes and pathways, which allow an organism to mount an orchestrated defense against toxic chemicals. Chemical defense genes may be especially important for early embryos, which must cope with the environment during sensitive stages of differentiation and development. Environmental chemicals handled by this defensome may include microbial products, heavy metals, phytotoxins and other natural compounds, and now, according to our new results, diatom PUAs such as decadienal. We can therefore hypothesize that the genes responsive to decadienal can be considered as part of the chemical defensome or stress surveillance system of the sea urchin *P. lividus*, affording protection from environmental toxicants. Our results also confirm sea urchin embryos as valid candidates for the study of stress and defense mechanisms in marine invertebrates.

By way of a coda, our results have important implications for understanding the cellular mechanisms underlying the responses of benthic organisms to aldehyde exposure. Sea urchins may come into contact with diatom PUAs in the field at the end of a bloom, with the mass sinking of diatoms to the sediment. Since they are browsing animals that eat phytoplankton and organic matter in the sand or mud, sea urchins may accumulate PUAs through feeding or be exposed to high local concentrations of these compounds that may affect growth performance [49]. This is of considerable ecological relevance considering the importance of diatom blooms in nutrient-rich aquatic environments.

## Materials and Methods

### Ethics Statement

*Paracentrotus lividus* (Lamarck) sea urchins were collected from a location that is not privately-owned nor protected in any way, according to the authorization of Marina Mercantile (DPR 1639/68, 09/19/1980 confirmed on 01/10/2000). The field studies did not involve endangered or protected species. All animal procedures were in compliance with the guidelines of the European Union (directive 609/86).

### Embryo culture, decadienal exposure and morphological analysis

Adults sea urchins of the species *Paracentrotus lividus* were collected during the breeding season by our fishermen in the Gulf of Naples, and transported in an insulated box to the laboratory within 1 h after collection and maintained in tanks with circulating sea water until testing. To induce gamete ejection, sea urchins were injected with 0.2 ml of 0.2 M acetylcholine (Sigma-Aldrich) through the peribuccal membrane. Eggs were washed with filtered sea water (FSW) and kept in FSW until use. Concentrated sperm was collected dry and kept undiluted at +4°C until use. Sperm to egg ratios were 100∶1 for both controls and treated embryos.

Eggs were fertilized as described above and allowed to develop at 20°C in a controlled temperature chamber at 12∶12 light∶dark cycle. Before fertilization, eggs were incubated for 10 min in the presence of different concentrations of decadienal (0.15, 0.20, 0.25, 0.30 and 0.35 µg/ml) or FSW (control). Experiments were conducted in triplicate using three egg groups collected from three different females. After 48 hours of incubation, morphological malformations were determined for, at least, 200 plutei using a light microscope (Zeiss Axiovert 135TV) in comparison to the control, embryos in sea water without decadienal.

### RNA extraction and cDNA synthesis

About 30000 eggs in 200 ml FSW were treated for 10 min with 0.15, 0.20, 0.25, 0.30 and 0.35 µg/ml decadienal; eggs were then fertilized and collected at different developmental times. Samples (30000 eggs in 50 ml) were collected at 5, 9, 24 and 48 hours post fertilization (hpf) by centrifugation at 1800 rcf for 10 min in a swing out rotor at 4°C. The pellet was washed with phosphate buffered saline and then frozen in liquid nitrogen and kept at −80°C. Total RNA was extracted from each developmental stage using TRIzol (Invitrogen) according to the manufacter's instructions. Extraction with chloroform/isoamyl alcohol (24∶1) was performed following RNA precipitation by addition of glycogen and isopropyl alcohol. Contaminating DNA was degraded by treating each sample with DNase RNase-free kit (Roche) according to the manufacter's instructions. The amount of total RNA extracted was estimated by the absorbance at 260 nm and the purity by 260/280 and 260/230 nm ratios, by Nanodrop (ND-1000 UV-Vis Spectrophotometer; NanoDrop Technologies). The integrity of RNA was evaluated by agarose gel electrophoresis. Intact rRNA subunits (28S and 18S) were observed on the gel indicating minimal degradation of the RNA. For each sample, 600 ng of total RNA extracted was retrotranscribed with iScript™ cDNA Synthesis kit (Biorad), following the manufacter's instructions. cDNA was diluted 1∶2 with H_2_O prior to use in Real Time qPCR experiments.

### Gene expression by Real Time qPCR

For all real time qPCR experiments the data from each cDNA sample were normalized using ubiquitin mRNA as endogenous control level, whose level remained relatively constant in all the developmental stages examined according to Nemer et al. ([50]; for more details see [17]). We analyzed *hsp70*, previously analyzed by Real Time qPCR in Romano et al. [17] and another sixteen new genes: heat shock protein 60 (*hsp60*), heat shock protein 56 (*hsp56*), 14-3-3 epsilon protein (*14-3-3ε*), metallothionein (*MT*), nectin (*Nec*), hatching enzyme (*hat*), SRY (sex determining region Y)-box 9 (*sox9*), cytochrome b (*cytb*), univin (*uni*), blastula protease 10 (*BP10*), p38 MAP kinase (*p38 MAPK*), DNA-methyltransferase (M*Tase*), glutamine synthetase (*GS*), spicule matrix protein 50 (*SM50*), spicule matrix protein 30 (*SM30*) and BMP5-7 (see Table S1). The gene sequences were retrieved from NCBI (http://www.ncbi.nlm.nih.gov). For each gene, specific primers were designed on the basis of nucleotide sequences (see Table 1): only for three genes we used primers reported in the indicated references.

**Table 1:** Accession numbers, primer sequences and length of PCR amplified fragments are reported for the genes analyzed.

Gene		Accession	Primer	Sequence	PCR fragment
		number		(5′ = >3′)	(bp)
**Heat shock protein 70**	*hsp70*	X61379	Pl_hsp70_Up	CAGAACCACGCCCAGCTATG	150
*(Romano et al., 2011)*			Pl_hsp70_Rev	GCTTGGATGCTACTATCGTTG	
**Heat shock protein 60**	*hsp60*	AJ249625	Pl_hsp60_F1	GAATATCCAGTGTACTCCGAC	160
			Pl_hsp60_R1	GCATCAGCTAAGAGGTCAAC	
**Heat shock protein 56**	*hsp56*	DQ464426	Pl_hsp56_F1	GGAGCTATGCTAAGGACATC	183
			Pl_hsp56_R1	CTACAGCCTTAGCGACAGTG	
**Cytochrome b**	*cytb*	M16528	Pl_Cyt_F1	GGGATACGTATTAGTCTGAGG	144
			Pl_Cyt_R1	CGAGTTAGGGTGGCATTGTC	
**Metallotionein**	*MT*	AJ310190	Pl_Mt_F1	GAAAGCAGTGTCCCTGTGCAG	162
			Pl_Mt_R1	CATGTACAGTTCCCTTCAGTG	
**Hatching enzyme**	*hat*	X65722	Pl_Hat_F1	CCACTGTGCATAATGTCCCG	138
			Pl_Hat_R1	CAGAGACAGGAGTCAGTAGAC	
**Blastula protease 10**	*BP10*	X65721	Pl_Bp10_F1	CTACGGGTGATCAGAAGGAG	156
			Pl_Bp10_R1	CTTCAGTGAGCATCATGTCTC	
**14-3-3 epsilon protein**	*14-3-3e*	AJ493680	Pl_Eps_F1	CGGATAGATACAATGACATGG	146
			Pl_Eps_R1	GCTGACTGTATGCAATGCTG	
**p38 mitogen-activated**	*p38 MAPK*	AM295340	Pl_p38_F1	GTGATCAGCTTGCTTGACTG	144
**protein kinase**			Pl_p38_R1	GTAGATGAGGAACTGGACGTG	
**Glutamine synthetase**	*GS*	L32699.1	Pl_GS_F1	GTGTCGGACCGATATCTGAC	177
			Pl_GS_R1	CTCCGATTGATCCGTACTCG	
**DNA-methyltransferase 1**	*MTase*	AJ418711	Pl_Met1_F1	GATCTCGTCAGACGATAGAAG	175
			Pl_Met1_R1	CTCTTGCTGTGTTAGCATTG	
**SRY (sex determining**	*sox9*	AY776326	Pl_Sox9_F1	GAGCTTCATCACTCCCTGTC	175
**region Y)- box9**			Pl_Sox9_R1	GATGGATGGAGAGAACTGCG	
**Nectin**	*Nec*	AJ578435	Pl_Nec_F1	CAAGCACAGCTGGGAATGG	158
			Pl_Nec_R1	GGTCATTTGTTCTTGCACTC	
**Univin**	*uni*	AJ302364	Pl_Uni_F1	ACTGGATCATCGCTCCGATG	259
*(Zito et al., 2003)*			Pl_Uni_R1	CATCGGCATCCACAAGCTTC	
**Spicule matrix protein 30**	*SM30*		Pl_SM30_F1	TTGGGTTCAGTTGGAGAACC	384
*(Zito et al., 2003)*			Pl_SM30_R1	GTTTCGTTGTCTTCGGGGTA	
**Spicule matrix protein 50**	*SM50*	AJ515510	Pl_Sm50_F1	GATGGCACACCAGCTTATCC	162
			Pl_Sm50_R1	CTGACGCTTCATGACTGGAG	
**Bone morphogenetic**	*BMP5-7*	AJ302363	Pl_BMP_F1	TGGCAGGAATGGATCATCGC	117
**protein 5–7**			Pl_BMP_R1	GAGTGTCTGCACGATGGCGTG	
*(Zito et al., 2003)*					

References are reported for the genes previously analyzed.

The amplified fragments using Taq High Fidelity PCR System (Roche) were purified from agarose gel using QIAquick Gel extraction kit (Qiagen) and specificity of PCR products for the sixteen genes was checked by DNA sequencing.

Specificity of amplification reactions was verified by melting curve analysis. The efficiency of each primer pair was calculated according to standard methods curves using the equation E = 10^−1/slope^. Five serial dilutions were set up to determine Ct values and reaction efficiencies for all primer pairs. Standard curves were generated for each oligonucleotide pair using the Ct values versus the logarithm of each dilution factor. PCR efficiencies were calculated for control and target genes and were found to be 2. Diluted cDNA was used as a template in a reaction containing a final concentration of 0.3 µM for each primer and 1× FastStart SYBR Green master mix (total volume of 10 µl). PCR amplifications were performed in a ViiA™ 7 Real Time PCR System (Applied Biosystems) thermal cycler using the following thermal profile: 95°C for 10 min, one cycle for cDNA denaturation; 95°C for 15 sec and 60°C for 1 min, 40 cycles for amplification; 72°C for 5 min, one cycle for final elongation; one cycle for melting curve analysis (from 60°C to 95°C) to verify the presence of a single product. Each assay included a no-template control for each primer pair. To capture intra-assay variability all Real Time qPCR reactions were carried out in triplicate. Fluorescence was measured using ViiA™ 7 Software (Applied Biosystems). The expression of each gene was analyzed and internally normalized against ubiquitin using REST software (Relative Expression Software Tool) based on Pfaffl method [51], [52]. Relative expression ratios above two cycles were considered significant. Experiments were repeated at least twice. Statistical analysis was performed using GraphPad Prism version 4.00 for Windows (GraphPad Software, San Diego California USA).

## Supporting Information

Table S1 **Function for the genes analyzed in the present study.**
(XLS)

Table S2 **Data of expression level are reported as a fold difference from control at 5, 9, 24 48 hpf.**
(XLS)

Table S3 **Data of expression level are reported as a fold difference from control at 5, 9, 24 48 hpf at decadienal concentrations of 0.15, 0.20, 0.25, 0.30, 0.35 *μ*g/ml.**
(XLS)
